# Complement Evasion Protects FCoV from Virus Clearance Within Prototypic FIP Lesions

**DOI:** 10.3390/v16111685

**Published:** 2024-10-29

**Authors:** Anne Hönl, Sandra Felten, Katharina Erber, Michèle Bergmann, Sven Reese, Regina Hofmann-Lehmann, Marina L. Meli, Andrea M. Spiri, Katrin Hartmann, Kaspar Matiasek

**Affiliations:** 1 LMU Small Animal Clinic, Centre for Clinical Veterinary Medicine, LMU Munich, 80539 Munich, Germany; michele.bergmann@lmu.de (M.B.); hartmann@lmu.de (K.H.); 2 Section of Clinical and Comparative Neuropathology, Institute of Veterinary Pathology, Centre for Clinical Veterinary Medicine, LMU Munich, 80539 Munich, Germany; erber@patho.vetmed.uni-muenchen.de; 3 Department of Small Animal Medicine, Center for Clinical Veterinary Medicine, Vetsuisse Faculty, University of Zurich, CH-8057 Zurich, Switzerland; sandra.felten@uzh.ch; 4 Section of Anatomy, Histology and Embryology, Faculty of Veterinary Medicine, LMU Munich, 80539 Munich, Germany; sven.reese@lmu.de; 5 Clinical Laboratory, Department of Clinical Diagnostics and Services, and Center for Clinical Studies, Vetsuisse Faculty, University of Zurich, CH-8057 Zurich, Switzerland; rhofmann@vetclinics.uzh.ch (R.H.-L.); mmeli@vetclinics.uzh.ch (M.L.M.); aspiri@vetclinics.uzh.ch (A.M.S.)

**Keywords:** FIP, complement system, immunohistochemistry, C1q, C9, CD46, CD59, immune evasion

## Abstract

Feline infectious peritonitis (FIP) is a fatal disease in cats caused by infection with feline coronavirus (FCoV). Despite severe inflammatory changes, defense mechanisms fail to achieve virus clearance. Some studies focused on various immune evasion mechanisms, but none of these studies elucidated the inefficacy of the complement system, which is one major player in FIP-associated immune pathogenesis. This study aimed to investigate the involvement of complement-regulating factors (CRFs). CRFs help modulate the immune response and prevent host tissue damage. Archived tissue samples from 31 deceased, FIP-affected cats were evaluated using multiplex immunohistochemistry for the spatial expression of the complement-regulating factors CD46 and CD59 in association with FIP lesions and their colocalization with complement-activating factor C1q and membrane attack complex C9 in relation to the presence and proximity of FCoV-infected cells. The FIP lesions of all 31 cats exhibited marked expression of both complement-regulating factors in proximity to FCoV-infected macrophages. Moreover, their expression in all 31 animals was significantly lower than the expression of the complement-activating factors C1q and C9 compared to areas farther distal to FCoV-infected cells. In conclusion, FCoV-infected macrophages in cats with FIP appear to use autocrine and paracrine expression of complement-regulating factors in their immediate environment to shield themselves from destruction by the complement system.

## 1. Introduction

Feline infectious peritonitis (FIP) is a fatal disease in cats caused by infection with feline coronavirus (FCoV). The virus infects and replicates in enterocytes. Upon mutation, it can infect and sustainably replicate in macrophages to cause FIP [1]. In this process, infected macrophages bypass immune system attack mechanisms [2], although they trigger extensive inflammation with complement activation [3]. The complement system plays an important role in the inflammatory response and the host’s defense against pathogens [4]. Three pathways of complement activation lead to the formation of the key enzyme C3/C5 convertase. This enzyme cleaves complement factors C3 and C5 into C3a, C3b, C5a and C5b. C3b labels antigens for phagocytes. The attachment of C5b to the cell membrane results in the activation of the membrane attack complex (MAC), in which complement factors C6–C9 create pores within the membrane of infected cells, which culminates in the lysis of the antigen-bearing cells [5]. Several factors may be recruited to prevent these processes. Among other proteins, the membrane cofactor protein (MCP) CD46 is a potent complement regulatory protein that protects host cells. It serves as a cofactor for serine protease factor I and inactivates C3b and C4b, which are linked to host cells, to prevent phagocytosis [6]. Farther downstream, CRF CD59 inhibits MAC formation by binding to C8, which prevents C9 from attaching to the complex [7]. As a result, the insertion of porins fails, which protects the cell from lysis.

Factors that activate the complement system, such as C1q, are the major opponents of CRFs. At the very beginning, C1q is part of the first complement complex. It can be activated by antibody-dependent and antibody-independent mechanisms and triggers cells equipped with membrane-bound C1q receptors. Once activated, it initiates the classical complement cascade [8]. C1q on its own also has complement-independent functions, such as recognition of cell debris and regulation of multiple cellular processes by interacting with a variety of cell surface molecules [9]. In addition to recruiting immune cells, the complement system itself may activate its major effector molecule, C5b9, which, as described above, leads to pores in the infected cells and ensures their lysis [10].

Several studies focused on the immune evasion of viruses involving the complement system and how they can be used in an artificial system to demonstrate their protecting activity. For example, the GP64 protein of the baculovirus, which is responsible for entry into the host cell, was recombined with the human decay accelerating factor (CRF). As a result, the baculovirus became resistant to inactivation by the complement system [11]. Moreover, cells infected with infectious bronchitis virus (IBV), a gamma coronavirus such as FCoV and a highly infectious avian pathogen, are protected from antibody-dependent complement-mediated lysis by CRF CD59 [12]. Another example to demonstrate the evolutionary adaptation of viruses to the complement system is HIV. It was shown that HIV viral preparations lacking CD55 (another CRF) and CD59 were very sensitive to complement and cell lysis occurred. In contrast, primary virus isolates from HIV patients were resistant to the complement system. Investigations showed that CD55 and CD59 of the host cell were inserted into the virion, which suggests that the virus protects itself from cell lysis through these CRFs [13]. Other viruses such as poxviruses are able to express a protein similar to complement regulatory proteins on their own [14].

The present study hypothesized that infected macrophages in cats with FIP inhibit the complement system by expressing complement regulatory factors, such as CD46 and CD59, to bypass the immune system. To address this hypothesis, the expression of CRFs and key players of the complement cascade was evaluated in relation to their localization and FCoV-infected cells in FIP lesions of 31 cats confirmed using RT-qPCR and/or immunohistochemistry.

## 2. Materials and Methods

### 2.1. Tissue Selection and Case-Specific Metadata

Archived samples of prototypic fibrinonecrotic and pyogranulomatous FIP lesions from 31 cats that died or were euthanized during the course of FIP from 2012 to 2021 were included in this study (Table S1).

The case collection included 11 European shorthair cats (35.5%), 5 British shorthair cats (16.1%), 4 Siamese cats (12.9%), 4 Main Coon cats (12.9%) (4/11), 3 sacred cats of Burma (6.5%), 1 Bengal cat (3.2%), 1 Siberian Forest cat (3.2%), 1 European longhair cat (3.2%), 1 Thai cat (3.2%) and 1 (3.2%) mixed breed cat (3.2%). The age ranged from 2 to 120 months (median 17.2 months) (29/31). The ages of two cats (2/31) were unknown. Eleven cats (35.5%) were female (5 of them neutered), and 20 cats (64.5%) were male (6 of them neutered). All of the cats were subjected to full necropsy, and FIP was confirmed via prototypical histopathological lesions plus positive immunohistochemistry (IHC) for FCoV antigen (clone FIPV3-70; Bio-Rad, 85622 Feldkirchen, Germany) within tissue macrophages and RT-qPCR for FCoV RNA whenever possible. For the control samples, see below.

### 2.2. Confirmative Testing

Archived FIP-positive formalin-fixed paraffin-embedded (FFPE) tissue samples were tested again for FCoV using IHC. The FFPE blocks were chilled on a −20 °C cooling panel, after which 2 µm thick serial sections were obtained using a microtome. The slides were mounted on standard histology slides (STAR FROST^®^, Waldemar Knittel Glasbearbeitungs GmbH, 38114 Braunschweig, Germany) and dried overnight in an incubator at 40 °C. After deparaffinization in xylene (20 min, room temperature (RT)), antigen retrieval was performed via microwave treatment in Tris-EDTA buffer (pH 9) at 800 W for 20 min. To block endogenous peroxidases, the slides were placed in 3% H_2_O_2_ solution for 15 min at RT. Before and between all of the following steps, the sections were washed by rinsing and immersion in Tris-buffered saline (TBS). Before pAB incubation, normal serum was applied to the slides for 30 min at RT. The slides were first incubated with FCoV antibody (MCA2194) at a dilution of 1:500 for 1 h at RT. The horseradish peroxidase (HRP)-conjugated polyclonal rabbit anti-mouse immunoglobulin antibody (DAKO, P0260, CA 95052, United States) was applied at a dilution of 1:100 and incubated for 50 min at RT. After the removal of the serum, the samples were stained with diaminobenzidine tetrahydrochloride (DAB; ImmPACTTM DAB^®^; Vector Laboratories, INC.; CA 94560, United States) under visual control. Finally, counterstaining was performed with hematoxylin, and the sections were dehydrated in an ascending alcohol series and xylene. The samples were coverslipped with xylene-based medium (EUKITT^®^, Plano GmbH; 35578 Wetzlar, Germany).

RT-qPCR analysis was performed on the tissue wherever DNA preservation was adequate to support the IHC results. For PCR analysis, 10 FFPE microtome shavings per tissue block were collected and transferred to sterile Eppendorf cups for further processing. Viral nucleic acids were isolated using the RNeasy^®^ FFPE Kit (Qiagen, Germantown, MN 20874, USA) and xylene for deparaffinization according to the manufacturer’s instructions. Negative controls (200 µL of phosphate-buffered saline (PBS; Gibco, Life Technologies Ltd., PA4 9RF Paisley, RFW UK)) were run in parallel to check for cross-contamination. FCoV RNA was amplified using an FCoV 7b gene RT-qPCR assay, as previously described [15].

### 2.3. Immunohistochemical Double Staining

Upon immunohistochemical confirmation of intralesional FCoV-infected macrophages, further sections were used for evaluations of CRF and the complement factors C1q and C9. All double staining started with labeling of the FCoV antigen (see above), followed by the complement regulatory or activating factors. IHC was performed on deparaffinized slides using single- and double-staining methods. An indirect method using HRP and DAB (ImmPACTTM DAB^®^; Vector Laboratories, INC.; Newarc, CA 94560, USA) and HistoGreen (HISOPRIME^®^; Biozol Diagnostica Vertrieb GmbH; 85386 Eching, Germany) as chromogens was used for signal detection. Sera were incubated in a humid chamber at RT. Normal serum (depending on the species in which the second antibody was generated) was diluted with blocking buffer, as described in the supplements, containing 1:20 avidin (Avidin/Biotin Blocking Kit, SP-2001). All specific antibodies were diluted in blocking buffer enriched with 2.5% serum from the species in which the secondary antibody was generated and 1:20 biotin (Avidin/Biotin Blocking Kit, SP-2001). The primary antibodies (pABs) were replaced with nonrelevant antibodies, and TBS was used as a negative control. The primary and secondary antibodies used for double IHC are listed below (Table 1). Each run included combined and separate stains of all primary antibodies. After the FCoV antigen was labeled, the slides were treated with LinBloc^®^ solution (Linaris, Biological Products; Biozol Diagnostica Vertrieb GmbH; 85386 Eching, Germany) 2 times for 2 min at RT, as described elsewhere [16], to remove bound primary and secondary antibodies from the tissues while leaving the DAB complex. Thereafter, the sections were directly covered with normal serum or treated with protease K (C1q) or microwaved in citrate buffer (pH 6.0) for 20 min (CD46, CD59). After 30 min at RT, the tissues were incubated overnight with the respective primary complement-associated antibody at 4 °C. The next day, the goat anti-rabbit antibody dilution was applied for 50 min at RT. The sections were incubated with avidin–biotin complex for 30 min and stained with HistoGreen (HISTOPRIME^®^, Biozol Diagnostica Vertrieb GmbH; 85386 Eching, Germany). Finally, counterstaining was performed with hematoxylin, and the sections were dehydrated in an ascending alcohol series and xylene. The samples were coverslipped with xylene-based medium (EUKITT^®^; Plano GmbH; 35578 Wetzlar, Germany). The second part of the double immunohistochemistry is also described for each second primary antibody separately in Table 1.

### 2.4. Microscopic Algorithm

For each set of stains, successful staining was verified by first checking single-labeled sections and then comparing the staining quality and intensity to the double-labeled sections. After the quality check, the examination focused on FCoV-positive areas, and the number of immunopositive cells was evaluated using the scores depicted in Table 2. After this assessment, the expression of complement-associated markers was evaluated with respect to their intensity (0–3) and proximity to FCoV expression from intrafocal via marginal to extrafocal areas. Intrafocal immunopositivity was considered co-expression or co-localization, and restriction to margins or more distant areas was considered intimate avoidance without a halo or with a clear zone of >2 macrophage diameters surrounding the aggregation of FCoV-positive cells.

### 2.5. Statistical Evaluation

Statistical analysis was performed using SPSS^®^ software(Version 29.0.1.0). Frequency tables were prepared, and all parameters were evaluated in relation to FCoV-positive cells. To determine the correlation between FCoV-positive cells and different CRFs and complement-activating factors, the Kendall tau correlation coefficient was used. A value of −1 indicates a perfect negative association, a value of 0 indicates no association, and a value of 1 indicates a perfect positive association [17]. Crosstables were generated to determine whether there was a correlation between the pathological changes and the staining patterns. P values were calculated to demonstrate statistical significance (*p* ≤ 0.05).

## 3. Results

### 3.1. Spatial Pattern and Yield of Feline Coronavirus-Infected Cells

A total of 54.8% (17/31) of the cats had >3 hotspots with more than 30 infected macrophages per focus in the tissues. Strong expression of the FCoV antigen but with fewer hotspots and 10–30 infected macrophages per lesion was detected in 38.7% (12/31) of the cats. Only 6.5% (2/31) of the lesions contained only a small number (<10) of infected macrophages.

### 3.2. Expression of Complement-Activating Factors Versus Complement-Regulating Factors

A narrow immunonegative fringe between FCoV-positive cells and C1q was observed in 51.6% (16/31) of the cats, and a wide fringe was detected in 48.4% (15/31) of the cats.

For C9, 32.3% (10/31) of the tissues had a narrow immunonegative fringe, and 67.7% (21/31) had a wide immunonegative fringe in relation to the FCoV-infected cells. Colocalization of C1q and C9 with FCoV-infected cells was not observed in any of the cases.

**Figure 1:**
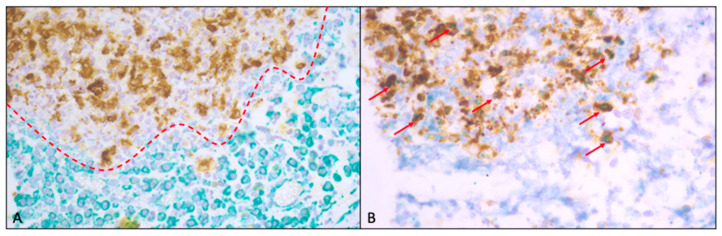
(**A**): Example of double IHC for FCoV and C1q showing sparse complement factor production (red line) in proximity to FCoV-infected macrophages. (**B**): Double IHC for FCoV and CD59, on the other hand, reveals coexpression and/or colocalization (red arrows) of CRF- and FCoV-infected macrophages. Both images were taken from the same lesion of the lung of one of the cats.

For CD46 expression, 74.2% (23/31) exhibited weak expression and/or sporadic colocalization with FCoV-infected macrophages, and 25.8% (8/31) exhibited strong immunopositivity and/or predominant colocalization with FCoV-infected macrophages. CD59 expression in 54.8% (17/31) of the tissues was weak and/or only sporadically colocalized and, in approximately 45.3% (14/31), was strongly expressed and/or predominantly colocalized to FCoV-infected macrophages. Figure 1 shows a representative example of the double IHC staining.

### 3.3. Comparative Staining Pattern: Complement-Regulating Versus Complement-Activating Factors

Complement-activating factors (CAFs) C1q and C9 were observed only in lesioned areas distant from FCoV-laden cells with at least one cell layer (one macrophage diameter) of immunonegativity. The negative correlation between FCoV-infected macrophages and C9 and C1q showed statistical significance (C1q: *p* ≤ 0.015; C9: *p* ≤ 0.027).

**Figure 2:**
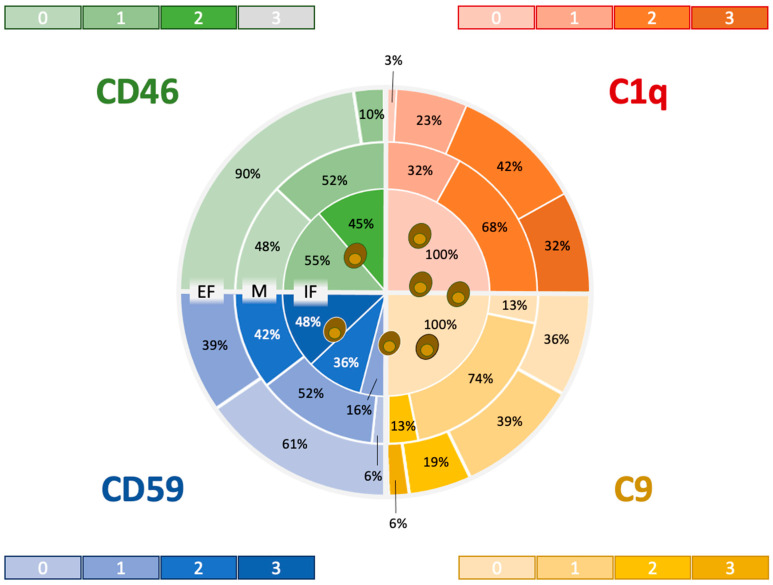
Heatmap showing the distribution and expression levels of complement-activating factors C1q and C9 and complement-regulating factors CD46 and CD 59 in relation to FCoV-infected macrophages: Expression zones included the intrafocal area (IF), which colocalizes with FCoV-expressing macrophages; the marginal zone (M), which comprises the layer next to the circumference of FCoV-expressing cells; and the extrafocal zone (EF), which is separated from the IF by an immunonegative fringe. The color intensity mirrors the immunohistochemical scores (“the darker, the stronger”), whereas the percentages indicate the yield of lesions that expressed the markers at the respective strength per zone. Notably, high expression scores in the CRF were concentrated around FCoV-infected cells, whereas high expression scores were detected in the CAF in the outer zones only.

The depicted CRFs CD46 and CD59 were expressed intimately in FCoV-positive cells only (Figure 2). Therefore, there was a significant correlation between CD46, CD59 and FCoV expression (CD46: *p* ≤ 0.004; CD59: *p* ≤ 0.001).

The staining was classified into intrafocal, marginal and extrafocal regions around the FCoV lesions. With increasing distance from FCoV-positive cell aggregates, C1q (Kendall’s tau b: 0.727) and C9 staining scores (Kendall’s tau b: 0.505) increased significantly. In contrast, CD46 (Kendall’s tau b: −0.724) and CD59 (Kendall’s tau b: −0.726) staining became significantly weaker. Therefore, the colocalization and coexpression of complement regulatory factors were concentrated around FCoV-infected macrophages, and no C1q-mediated complement activation or expression of the membrane attack component C9 was detected in proximity to FCoV-infected macrophages. No expression of CRFs was detected. Correlations of C1q, C9, CD46 and CD59 with FCoV-infected cells are depicted in Supplementary Figures S1–S4.

## 4. Discussion

It has been widely accepted that FIP-causing FCoV uses a variety of strategies to evade the host immune response and establish an infection that leads to the pathological manifestation of FIP [18,19,20,21,22,23,24,25]. This study is the first to focus on the spatial relationship between FCoV antigen expression and the expression of complement-regulating factors as a possible trigger of immune evasion in lesions caused by FIP. Consistent with the hypothesis that FCoV-infected macrophages protect themselves from the host immune system by expressing complement-regulating factors, the present study demonstrated that the coexpression and colocalization of the two CRFs CD46 and CD59 occurred with FCoV-infected macrophages. In contrast, the net expression of the complement factors C1q and C9 was observed only in cells distant from FCoV-infected cells. This finding leads to the conclusion that FCoV-infected macrophages ensure autocrine and paracrine expression of complement-regulating factors in their intimate environment during the progression of FIP and, thus, protect themselves against destruction by the otherwise deleterious effects of the complement system and complement-independent C1q actions. One study already demonstrated a role for CD46 and CD59 upregulation in human multiple myeloma, which limits the efficacy of immunotherapy by isatuximab [26]. In contrast, therapeutic suppression of CD46 expression and inhibition of CD59 by specific peptides render multiple myeloma treatment with daratumumab and isatuximab far more effective [27]. Whether these modulations resemble a strategy to increase the efficacy of the immune system as an ancillary treatment for cats with FIP, particularly highly pathogenic strains, must be evaluated.

For SARS-CoV-2 infection, it has been hypothesized that CD46 is involved in the entry of the virus into host cells via interactions with structural proteins [28]. CD55 and CD59, but not CD46, are involved in the hyperactivation and deposition of the complement factors C3, C3b/iC3b/C3d and C5b-9 in infected lung lesions of coronavirus disease 2019 (COVID-19)-affected people. The increased expression of CD55 and CD59 could represent a physiological feedback loop for self-protection from complement hyperactivation [29]. FCoV might take advantage of this loop because CD59 was also upregulated in FIP lesions. This expression was parallel to CD46. Whether CRFs are also incorporated into the virions as in HIV remains unclear [13]. FCoV-associated CRFs appear to inhibit lysis of infected feline cells and cause the downregulation of the complement system in infected areas. Consistent with this interpretation, the upstream complement activators C1q and C9, which are part of the downstream effector MAC, were essentially suppressed in areas close to FCoV-positive macrophages.

Consequently, complement-mediated lesions unequivocally spared FCoV-expressing foci, which can explain the failure of virus clearance in these areas. These effects were observed in all cats irrespective of age, breed and pathological subtypes of FIP.

FIP-associated FCoV has proven to interfere with various components of the immune system, including macrophages and T lymphocytes [30,31,32]. The pathological changes that occur in FIP could also be related to the reaction of macrophages to the viral infection and to the host immune system responding to infected cells. The immune evasion of FIP occurs at different levels.

Vermeulen et al. [33] reported a reduction in natural killer (NK) cells and regulatory T cells (Tregs; CD4+CD25+Foxp3+) in the peripheral blood, mesenteric lymph and spleen of cats with FIP. Lymph node-derived NK cells were also significantly less cytotoxic in cats with FIP compared to cats without FIP. Furthermore, regulatory CD4+CD25-Foxp3+ and CD3+CD8+Foxp3+ lymphocytes were reduced in the blood and lymph nodes of FIP-affected cats. The decrease in Tregs is likely decisive for the uncontrolled immune response and the inflammatory state in these cats. The findings of Vermeulen et al. [33] are similar to the immunopathology of SARS-CoV-1 and SARS-CoV-2. In humans with COVID-19, NK cells are significantly reduced in infected patients [34,35].

Takano et al. [31] demonstrated that the ratio of surface immunoglobulin-positive (sIG+) CD21- B cells was greater in cats with FIP than in specific pathogen-free (SPF) cats. Additionally, the number of cells expressing the plasma cell master gene B-lymphocyte-induced maturation protein 1 (Blimp-1) was increased in the peripheral blood of cats with FIP. The mRNA expression of IL-6, a CD40 ligand, and the B-cell-activating factor (BAFF) from the tumor necrosis factor family, all involved in B-cell differentiation and survival, was elevated in the macrophages of cats with FIP. Moreover, mRNAs encoding these B-cell differentiation/survival factors are overexpressed in antibody-dependent enhancement (ADE)-induced macrophages [31]. These data led to the conclusion that FCoV-infected macrophages overproduced B-cell differentiation and survival factors and, thereby, vigorously promoted B-cell differentiation into plasma cells that flooded the body with immunoglobulins to cause hypergammaglobulinemia. These immunoglobulins are ineffective for virus defense and appear to promote immunopathology. The impact of excessive immunoglobulins on the efficacy of the antiviral activities of the feline complement system versus complement-mediated immunopathology requires further elucidation. A broad understanding of the immunopathology of FIP opens an important window for therapies complementing the use of new nucleoside analogues, in particular of highly virulent strains. For example, the latest outbreak of FIP in Cyprus with a highly pathogenic canine/feline recombinant coronavirus has led to new challenges and will require detailed investigations of the pathogenesis, including variations among the different mutants [36]. There is a need for further research to determine whether this highly pathogenic strain utilizes other immune evasion mechanisms. The results generated in the present study demonstrate that complement repression resembles another component of immune evasion strategies of FCoV in FIP cats. The CAF C1q was deliberately chosen to investigate the classical complement initiation pathway. Its suppression shows early interference that is not influenced by CD46 or CD59. For further elucidation of C1q suppression, it would be reasonable to screen “early” regulatory factors, such as C1-INH and C1qR, another protector against hyperactivation of the complement system, which appear to impact C1q directly [37,38].

One limitation of this study could be that the C9 component was used instead of activated C5b9 (MAC). However, tracing of the latter [10] in feline tissue requires cryostat sections [39] that are not available from the plethora of lesions in archived postmortem cases. Moreover, postmortem intervals, fixation times and sampling modes differed essentially in this collection of sporadically submitted FIP cases. Deeper insights into FIP immunolocalization, transcriptomics and proteomics require the application of standardized protocols for multimodal investigations. Furthermore, prospective sampling should consider the collection of intravital tissue, blood and effusion samples, such as described in a treatment study by the authors’ research group [40]. Systematic biobanking is a key player in research on immune escape and antiviral therapy.

## 5. Conclusions

This study showed that the complement-regulating factors CD46 and CD59 are involved in the pathogenesis of FIP. These factors are secreted by infected macrophages and surrounding cells in an autocrine–paracrine loop and can protect these cells and the contained virus from the host’s immune defense. This effect does not reach areas far beyond the infected areas. Therefore, the complement system remains a major player in the systemic immunopathology of FIP. Further CRFs likely play complementary roles and should be further investigated. The complexities of immune evasion in FIP present significant challenges. Elucidation of the mechanisms underlying FCoV immune evasion could pave the way for the development of targeted therapeutic interventions and preventive strategies. Continued research efforts aimed at unraveling the intricacies of FIP immunopathogenesis are essential for improving clinical outcomes and advancing feline health.

## Figures and Tables

**Table 1:** Protocols for staining of complement-associated factors.

	C1q	C9	CD46	CD59
Blocking-buffer	^1^IHC-buffer plus 2.5% goat serum			
Pretreatment	Protease K DAKO, S3020 Dilution: 1:100 8 min	-	Citrate buffer (pH 6) Microwave at 800 W 20 min	Citrate buffer (pH 6) Microwave at 800 W 20 min
Normal serum	Blocking buffer plus avidin (Avidin/Biotin Blocking Kit, SP-2001) (1:20) 30 min			
1. Antibody *	Blocking buffer with Biotin (Avidin/Biotin Blocking Kit, SP-2001) (1:20) plus Polyclonal Rabbit Anti-Human C1q Complement (DAKO, A 0136 P0260, CA 95052, USA) Dilution: 1:200 Overnight	Blocking buffer with Biotin (Avidin/Biotin Blocking Kit, SP-2001) (1:20) plus Rabbit Recombinant Monoclonal C9 (ab173302; abcam; Cambridge, CB2 0AX, UK) Dilution: 1:400 Overnight	Blocking buffer with Biotin (Avidin/Biotin Blocking Kit, SP-2001) (1:20) plus Rabbit Polyclonal CD46 antibody (ab231984; abcam; Cambridge, CB2 0AX, UK) Dilution: 1:100 Overnight	Blocking buffer with Biotin (Avidin/Biotin Blocking Kit, SP-2001) (1:20) plus Rabbit Polyclonal CD59 antibody (ab69084; abcam; Cambridge, CB2 0AX, UK) Dilution: 1:100 Overnight
2. Antibody *	Blocking buffer plus Goat Anti-Rabbit IgG, Biotinylated (Vector Laboratories, BA-1000) Dilution: 1:200 50 min			
Signal amplification	Streptavidin–biotin complex method (VECTASTAIN ABC Kit; Vector Laboratories)			
Staining	HistoGreen (HISTOPRIME^®^, Biozol Diagnostica Vertrieb GmbH; 85386 Eching, Germany)			

* Dilutions and protocols optimized in a pilot trial; ^1^IHC-buffer: 500 mL TBS; 1% Bovine serum albumin; 0.1% Triton X-100; 0.2% Gold Fish Gelatin; 0.02% sodium azide (1 g in 10 mL); pH: 7.4 (Table S2); TBS: Stock solution in a dilution 1:10 with distilled water (Table S2).

**Table 2:** Scoring system of FCoV*-positive cells and relation to their immunopositivity for C1q, C9, CD46 and CD59.

FCoV*-positive cells	
No immunopositive cells	0
Individual immunopositive cells	1
Individual aggregates of 10–30 cells per lesion	2
Multiple hotspots with >30 cells	3
Intrafocal, marginal and extrafocal immunostaining of CD46, CD59, C1q and C9 in lesions with FCoV*-positive cells	
No immunopositive signal in the described area	0
Some cells and slight coloration in the described area	1
Some cells with medium staining in the described area	2
Very many cells with high-grade staining in the area described above	3
FCoV*-positive cells in relation to CD46 and CD59	
No colocalization	0
Weak and/or sporadic colocalization with FCoV*	1
Strong and/or predominant colocalization with FCoV*	2
FCoV*-positive cells in relation to C1q and C9	
Separate expression with partly small or blurred distance (1–2 cell layers) to FCoV*-positive cells	1
Separate expression exclusively at a clear distance from FCoV*-positive cells	2

* Feline Coronavirus.

## Data Availability

The authors confirm that the datasets analyzed during this study are available from the corresponding authors upon reasonable request.

## Supplementary Materials

The following supporting information may be downloaded from https://www.mdpi.com/article/10.3390/v16111685/s1, Table S1: Signaling and clinical signs of the cats from which the samples were taken for this study; Table S2: Preparation of TBS and IHC buffers; Figure S1: Correlations between C1q- and FCoV-infected macrophages and the number of FCoV-infected macrophages and their location in lesions; Figure S2: Correlation between C9- and FCoV-infected macrophages in relation to the number of feline coronavirus-infected macrophages and their location in lesions; Figure S3: Correlation between CD46- and FCoV-infected macrophages in relation to the number of FCoV-infected macrophages and their location in lesions; Figure S4: Correlation between CD59- and FCoV-infected macrophages in relation to the number of FCoV-infected macrophages and their location in lesions.
