# Supplementary material for: Complement Evasion Protects FCoV from Virus Clearance Within Prototypic FIP Lesions
Source: Viruses. 2024 Oct 29;16(11):1685. doi: 10.3390/v16111685 (PMC11598988; doi:10.3390/v16111685)
Supplement: Supplementary file 1 [file viruses-16-01685-s001.zip › viruses-3278545-supplementary.pdf]

Table S1. Signalement and clinical signs of the cats from which the samples were taken for the study

Cat-Nr.	Age in month	Sex	Breed	Diagnosis of FIP[1]	Pathological signs
1	?	female	ESH[2]	IHC[5]	granulomatous changes without effusion
2	5	male	Siam	IHC[5]	granulomatous changes with effusion
3	3	male	BSH[3]	IHC[5] and RT–qPCR[6]	granulomatous changes and CNS manifestation without effusion
4	5	male neutered	Siberian Forest cat	IHC[5] and RT–qPCR[6]	granulomatous changes with effusion
5	9	female	Siam	IHC[5] and RT–qPCR[6]	granulomatous changes with effusion
6	5	male	Main Coon	IHC[5] and RT–qPCR[6]	granulomatous changes with effusion
7	42	male	BSH[3]	IHC[5] and RT–qPCR[6]	granulomatous changes without effusion
8	12	female	Holy Birma	IHC[5] and RT–qPCR[6]	granulomatous changes and CNS manifestation with effusion
9	12	male	ESH[2]	IHC[5] and RT–qPCR[6]	granulomatous changes without effusion
10	9	female	Siam	IHC[5] and RT–qPCR[6]	granulomatous changes with effusion
11	7	female	BSH[3]	IHC[5] and RT–qPCR[6]	granulomatous changes and CNS manifestation with effusion
12	5	male	Thai cat	IHC[5] and RT–qPCR[6]	granulomatous changes with effusion
13	12	male	Siam	IHC[5] and RT–qPCR[6]	granulomatous changes with effusion

14	8	male	BSH[3]	IHC[5] and RT–qPCR[6]	granulomatous changes with effusion
15	36	male neutered	ESH[2]	IHC[5] and RT–qPCR[6]	granulomatous changes and CNS manifestation with effusion
16	9	male neutered	ESH[2]	IHC[5] and RT–qPCR[6]	granulomatous changes and CNS manifestation with effusion
17	?	female	ESH[2]	IHC[5] and RT–qPCR[6]	granulomatous changes and CNS manifestation with effusion
18	2	male	ESH[2]	IHC[5] and RT–qPCR[6]	granulomatous changes and CNS manifestation with effusion
19	16	female neutered	ESH[2]	IHC[5] and RT–qPCR[6]	granulomatous changes with effusion
20	12	male neutered	ESH[2]	IHC[5] and RT–qPCR[6]	granulomatous changes with effusion
21	12	female neutered	ESH[2]	IHC[5] and RT–qPCR[6]	granulomatous changes and CNS manifestation with effusion
22	10	male	Main Coon	IHC[5] and RT–qPCR[6]	granulomatous changes with effusion
23	12	male neutered	BSH[3]	IHC[5] and RT–qPCR[6]	granulomatous changes and CNS manifestation with effusion
24	20	female neutered	ESH[2]	IHC[5] and RT–qPCR[6]	granulomatous changes with effusion
25	9	male	Holy Birma	IHC[5] and RT–qPCR[6]	granulomatous changes with effusion
26	120	female neutered	ESH[2]	IHC[5] and RT–qPCR[6]	granulomatous changes and CNS manifestation without effusion

27	72	male neutered	ELH[4]	IHC[5] and RT–qPCR[6]	granulomatous changes with effusion
28	5	male	Main Coon	IHC[5] and RT–qPCR[6]	granulomatous changes and CNS manifestation with effusion
29	18	male	Main Coon	IHC[5] and RT–qPCR[6]	granulomatous changes and CNS manifestation without effusion
30	7	male	Bengal	IHC[5] and RT–qPCR[6]	granulomatous changes and CNS manifestation with effusion
31	6	female	Mixed	IHC[5] and RT–qPCR[6]	granulomatous changes and CNS manifestation without effusion

[1] Feline infectious peritonitis; [2] European shorthair; [3] British shorthair; [4] European longhair; [5] Immunohistochemistry to label FCoV antigen; [6] Reverse transcription–polymerase chain reaction to detect FCoV RNA

Table S2: Preparation of TBS and IHC buffers

	Ingredients	Manufacturer details
**Stock solution**	2000ml desitlised water	
	121g TRIS	Article number: 1115KG001; neoFroxx GmbH; 64683 Einhausen, Germany
	90g NaCl (natriumchlorid)	Articel number: LC-5932.1; neoFroxx GmbH; 64683 Einhausen, Germany
	HCL 25% to get a pH of 7.6	Article number: LC-7038.2; neoFroxx GmbH; 64683 Einhausen, Germany
**TBS**	Stock solution in a dilution of 1:10 with desitlised water	
**IHC-buffer**	500ml TBS	
	1% Bovine serum albumin	Article number: A7906-100G; Merck; Sigma-Aldrich; Buenos Aires 1430; Argentina
	0.1% Triton X-100	Article Number: 37238; SERVA Feinbiochemica, Analytische Reagenzien; 69115 Heidelberg, Germany
	0.2% Gold Fish Gelatine	Article number: G7041-100G; Sigma-Aldrich; Buenos Aires 1430; Argentina
	0.02% natrium azid (1g in 10ml)	Article number: 6688.0250; Merck; Sigma-Aldrich; Buenos Aires 1430; Argentina

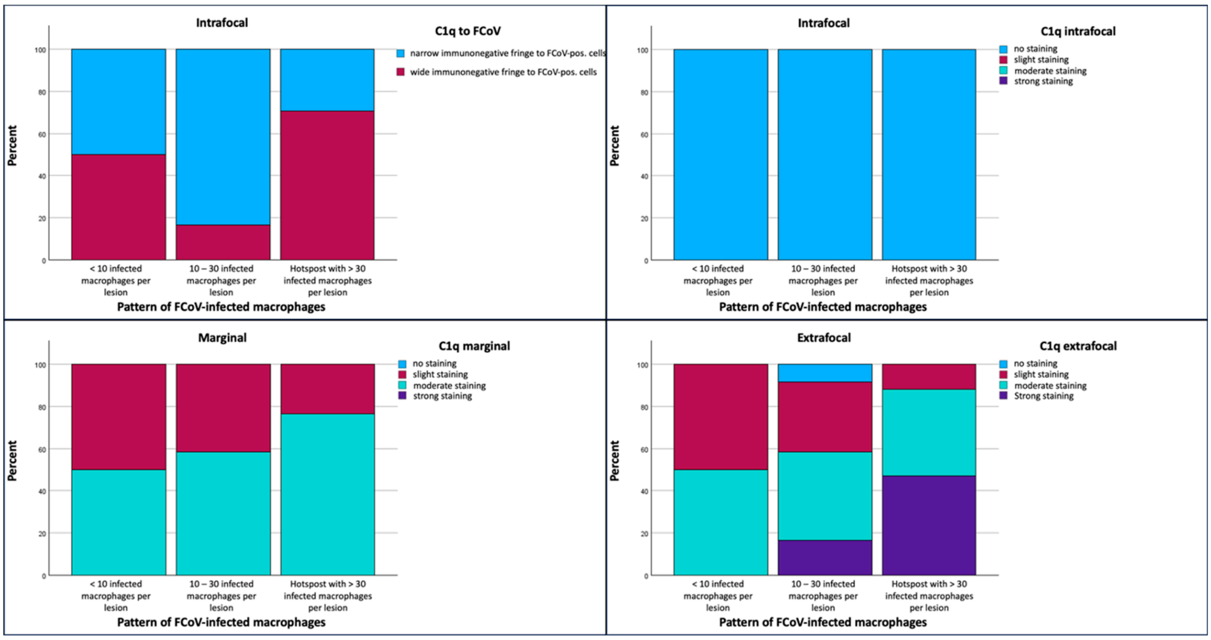

Figure S1: Correlations between C1q- and FCoV-infected macrophages and the number of feline coronavirus-infected macrophages and their location in lesions. **Top left:** the distance from C1q to FCoV-infected macrophages in relation to the number of FCoV-positive cells, narrow represents a distance of 1-2 cell layers and wide represents a distance of more than 2 cell layers. One cell layer is 1 macrophage diameter. **Top right:** focusses precisely on the staining intensity of C1q directly intrafocal. **Bottom left:** focuses on the staining intensity of the C1q margin. **Bottom right:** focuses on the intensity of C1q extrafocal staining. **C1q staining is essential:** Intrafocally, there is no positivity for C1q. The staining intensity increases with increasing distance to FCoV-positive cells ($p \leq 0.02$).

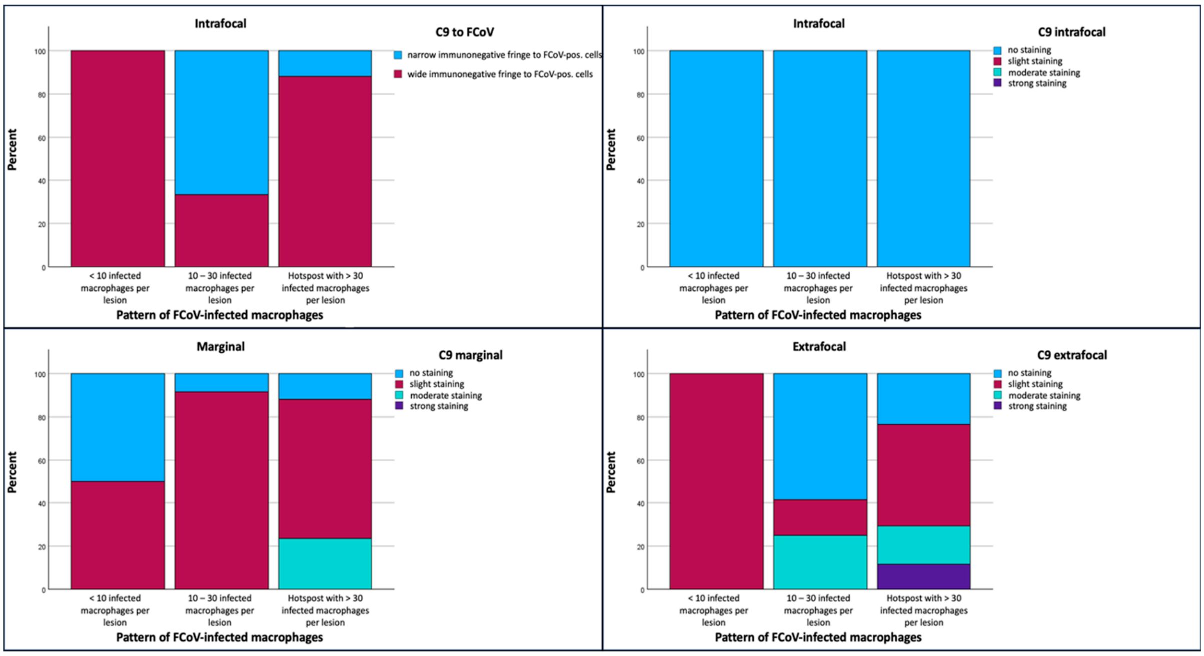

Figure S2: Correlation between C9- and FCoV-infected macrophages in relation to the number of feline coronavirus-infected macrophages and their location in lesions. **Top left:** the distance from C9 to FCoV-infected macrophages in relation to the number of FCoV-positive cells, narrow represents a distance of 1-2 cell layers and wide represents a distance of more than 2 cell layers. One cell layer is 1 macrophage diameter. **Top right:** focuses on the staining intensity of C9 directly intrafocal. **Bottom left:** focuses on the staining intensity of the C9 margin. **Bottom right**: focuses on the intensity of C9 extrafocal staining. **C9 staining is essential:** Intrafocally, there is no positivity for C9. With increasing distance to FCoV-positive cells, the positivity for C9 becomes stronger ($p \leq 0.03$) but not as strong as for C1q.

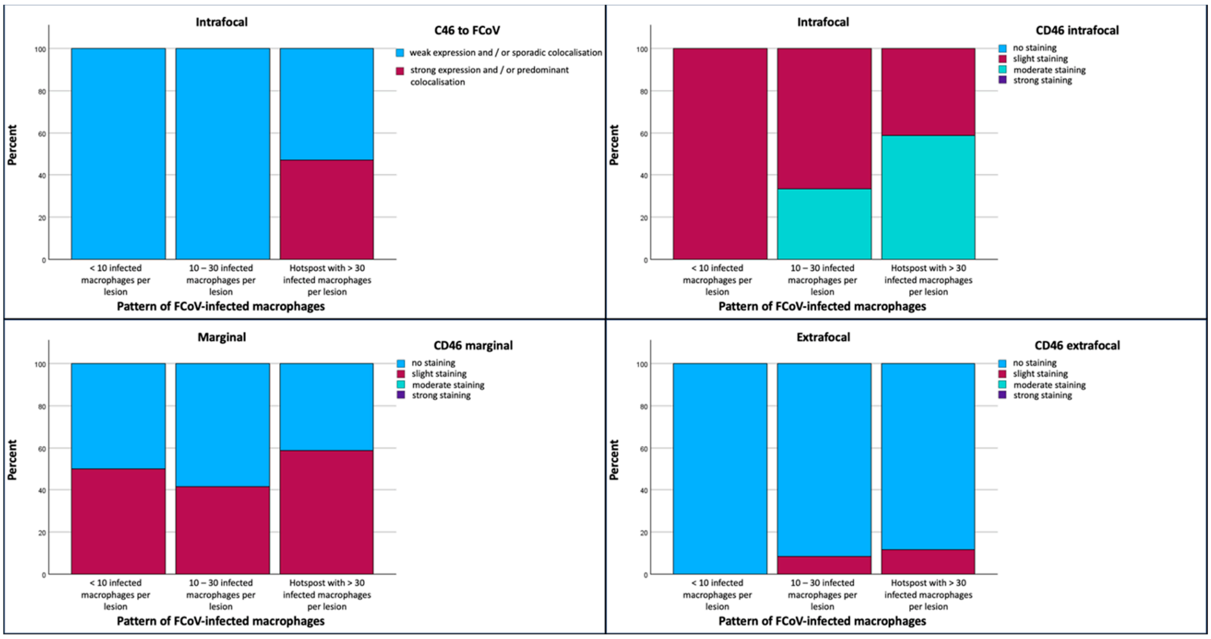

Figure S3: Correlation between CD46- and FCoV-infected macrophages in relation to the number of feline coronavirus-infected macrophages and their location in lesions. **Top left:** the expression and colocalization of CD46 in FCoV-infected macrophages in relation to the number of FCoV-positive cells. **Top right:** focuses on the intensity of CD46 staining directly intrafocal. **Bottom left:** focuses on the staining intensity of the CD46 margin. **Bottom right:** focuses on the intensity of CD46 staining. **C46 staining is essential:** CD46 is primarily expressed intrafocally ($p \leq 0.01$). Expression decreases with increasing distance to FCoV-infected cells. Hardly any CD46 is found extrafocally.

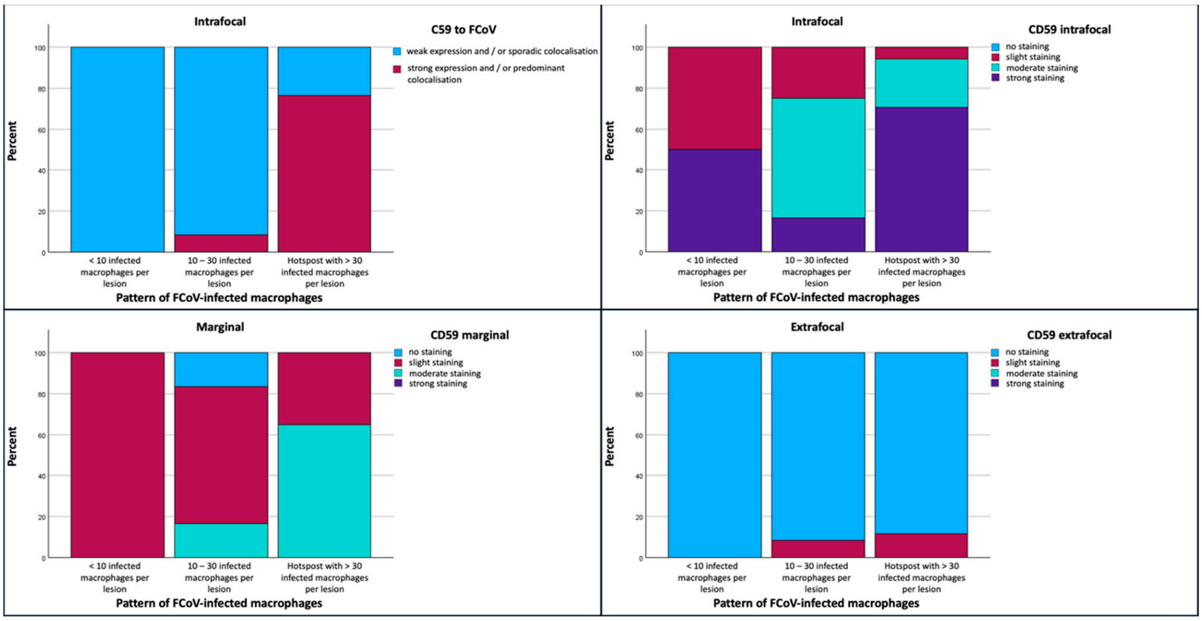

Figure S4: Correlation between CD59- and FCoV-infected macrophages in relation to the number of feline coronavirus-infected macrophages and their location in lesions. **Top left:** the expression and colocalization of CD59 in FCoV-infected macrophages in relation to the number of FCoV-positive cells. **Top right:** focuses on the intensity of CD59 staining directly intrafocally. **Bottom left:** focuses precisely on the staining intensity of the CD59 margin. **Bottom right:** focuses on the intensity of CD59 extrafocally. **C59 staining is essential:** CD59 is expressed primarily intrafocally ($p \leq 0.01$). Expression decreases with increasing distance to FCoV-infected cells. Hardly any CD59 is found extrafocally**.**
